# Artificial intelligence for posterior capsule opacification

**DOI:** 10.3389/fmed.2025.1695525

**Published:** 2025-12-12

**Authors:** Gurnoor Gill, David Taylor Gonzalez, Harshal Sanghvi, Mak Djulbegovic, Ayam Suleiman, Shailesh Gupta

**Affiliations:** 1 Charles E. Schmidt College of Medicine, Florida Atlantic University, Boca Raton, FL, United States; 2 Broward Health North, Deerfield Beach, FL, United States; 3 College of Business, Florida Atlantic University, Boca Raton, FL, United States; 4 Wills Eye Hospital, Philadelphia, PA, United States; 5 Advanced Research LLC, Deerfield Beach, FL, United States; 6 Speciality Retina Center LLC, Deerfield Beach, FL, United States

**Keywords:** artificial intelligence, posterior capsular opacification, decision support systems, deep learning, cataract surgery

## Abstract

Posterior capsule opacification (PCO) remains the most common long-term complication of cataract surgery, affecting up to one-fifth of patients within 5 years and often requiring neodymium: yttrium–aluminum–garnet (Nd:YAG) laser capsulotomy. Clinical decisions about if and when to intervene depend primarily on subjective assessments and carry non-trivial risks, including transient intraocular pressure spikes, cystoid macular edema, and rare retinal detachment. Recent advances in artificial intelligence (AI), spanning classical machine learning and deep convolutional neural networks, offer an objective, data-driven framework to (1) detect and grade PCO severity from imaging (retro-illumination photographs, OCT, Scheimpflug tomography), (2) stratify individual risk of clinically significant opacification and personalize follow-up, and (3) support timing and dosing of Nd:YAG capsulotomy. AI models have achieved expert-level performance (e.g., AUC up to 0.97 for binary detection of vision-threatening PCO, correlation r ≈ 0.83 for continuous severity scores, C-index ≈ 0.87 for capsulotomy risk nomograms), reducing observer bias and standardizing care. To address the “black-box” nature of complex models, mechanistic interpretability techniques, such as heatmaps and quantifiable feature extraction, are emerging to clarify decision logic and bolster clinician trust. Key challenges include assembling large, diverse, multi-center datasets (potentially via federated learning), prospective validation in real-world settings, regulatory approval, seamless integration into electronic health records and imaging workflows, and ensuring data privacy. Future directions emphasize true multimodal fusion of slit-lamp, OCT, and Scheimpflug tomography data, intraoperative feedback systems to minimize residual lens epithelial cells, patient-driven home monitoring via smartphone apps, and user-tunable AI thresholds to align with individual clinician and patient priorities. By combining transparent AI insights with surgical expertise, these tools can transform PCO management. They may optimize visual rehabilitation, minimize unnecessary procedures, and enhance safety in cataract postoperative care.

## 1 Introduction

Artificial intelligence (AI) is reshaping modern medicine by applying machine learning, deep learning, and natural language processing to analyze large, complex datasets and generate actionable insights (1–5). These systems detect subtle patterns in medical images, predict disease trajectories, and support diagnostics with accuracy comparable to experts (6–11). AI thrives in high-dimensional domains such as genomics, radiology, and pathology, and advances precision medicine by integrating multimodal data, from electronic health records to wearable sensors, to create personalized risk profiles and optimize treatment (12–15). This shift to predictive, preventive care may improve outcomes while reducing costs and enhancing efficiency (16, 17). AI also streamlines workflows, triaging images, generating preliminary reports, and flagging urgent findings to speed interventions in critical care areas (18–20). Embedded decision-support tools now provide point-of-care recommendations and federated learning and secure computation allow cross-institutional model training without compromising privacy (21–23). Yet, widespread adoption demands rigorous validation, regulatory oversight, and ethical governance to ensure transparency, accountability, and equity (24–26).

Building on its broad applications in medicine, AI has made particularly rapid and impactful strides in ophthalmology, a specialty uniquely suited to algorithmic analysis due to its reliance on high-resolution, image-rich diagnostic modalities (27, 28). Ophthalmic imaging technologies such as optical coherence tomography (OCT), fundus photography, and fluorescein angiography generate large volumes of structured and unstructured data that lend themselves to deep learning–based interpretation (28–30). AI models have demonstrated an enhanced level of detection for a wide range of ocular diseases, including diabetic retinopathy, age-related macular degeneration, glaucoma, retinopathy of prematurity, and corneal disorders (31). For example, convolutional neural networks have been trained to identify early, subtle pathological changes in OCT and fundus images, enabling earlier diagnosis and intervention compared to conventional screening methods (32, 33). FDA-approved autonomous AI systems, such as IDx-DR for diabetic retinopathy screening, exemplify how these tools can extend access to care in primary care and teleophthalmology settings, particularly in underserved regions where subspecialty expertise is limited (34).

Beyond diagnosis, AI is increasingly being integrated into prognostication, surgical planning, and treatment monitoring within ophthalmology (35, 36). Predictive models can estimate the risk of disease progression, forecast treatment response in conditions such as neovascular AMD, and guide personalized anti-VEGF injection schedules (37, 38). In refractive and cataract surgery, AI-driven biometry and intraocular lens power calculation tools have improved refractive accuracy, while machine learning–based anterior segment analysis supports optimal patient selection for procedures like phakic IOL implantation (39, 40). Additionally, natural language processing has been applied to large ophthalmology-specific datasets, enabling automated analysis of clinical notes and research literature to identify novel associations and streamline chart reviews (41–43). As the field moves toward precision ophthalmology, integrating AI with multimodal imaging, genetic data, and systemic health information holds the promise of creating holistic patient profiles that enhance both individualized care and population-level screening initiatives (44).

Despite the rapid expansion of AI applications in ophthalmology, one area that remains relatively underexplored in the literature is its role in predicting and managing post-surgical complications (45). While most current AI research has focused on preoperative diagnostics and intraoperative guidance, the postoperative period represents a critical phase where timely detection and intervention can significantly influence long-term visual outcomes (46–48). Surgical procedures such as cataract extraction, corneal transplantation, glaucoma filtration surgery, and retinal detachment repair all carry distinct risk profiles for complications ranging from infection and inflammation to refractive surprises and secondary ocular pathologies (48). Yet, there is a paucity of robust AI models trained specifically on postoperative datasets to anticipate these adverse events or to detect early, subtle signs of their onset (49, 50). This gap is partly due to challenges in collecting high-quality, standardized postoperative data across diverse populations and follow-up intervals, as well as the inherent variability in surgical techniques and patient adherence to postoperative care regimens (51–53). One compelling example where such an approach could be transformative is in the prediction, early detection, and management of posterior capsular opacification (PCO), the most common delayed complication following cataract surgery.

**FIGURE 1:**
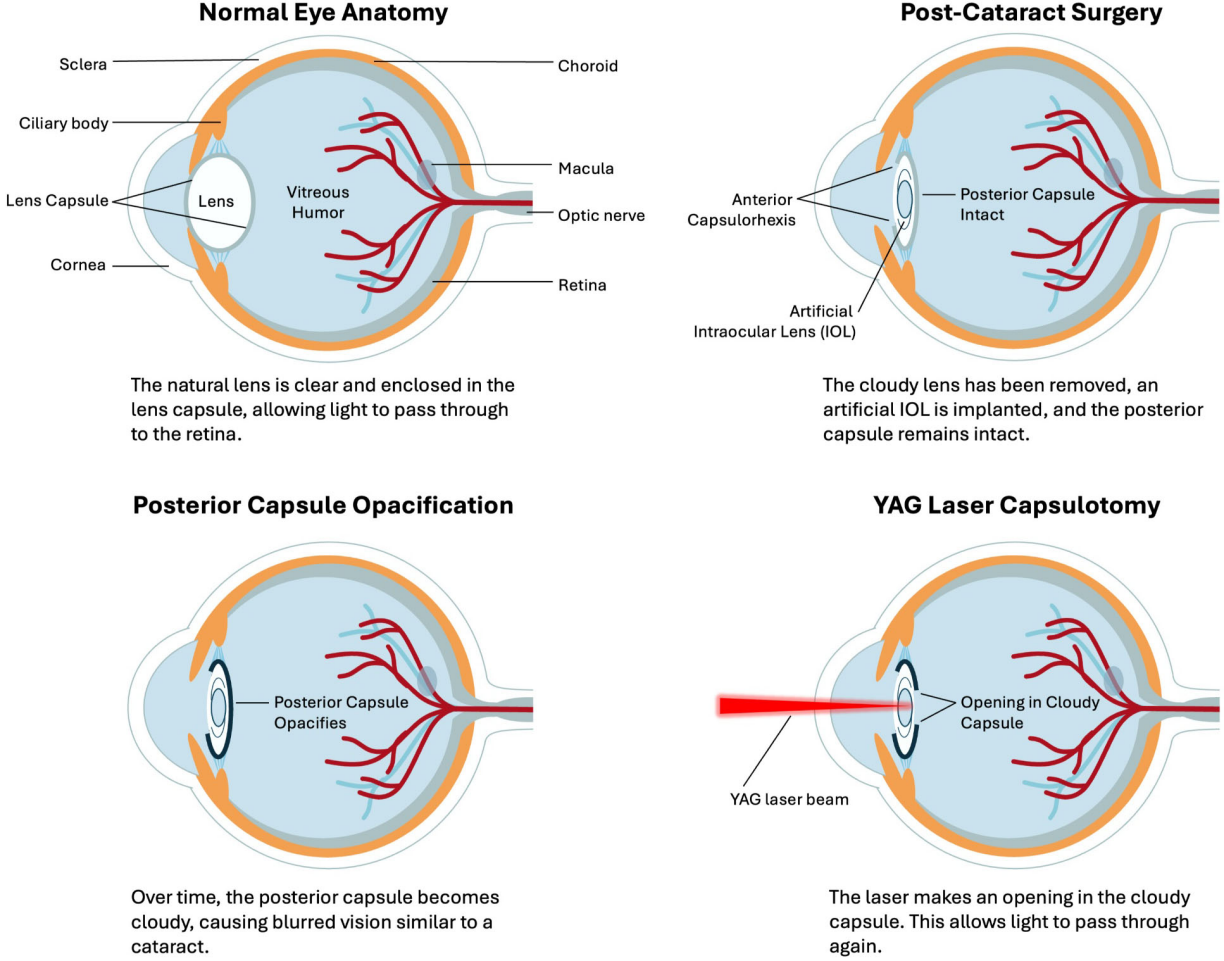
Schematic representation of posterior capsule opacification and its management. (Top left) Normal eye anatomy with a clear natural lens enclosed in the capsule. (Top right) After cataract surgery, the cloudy lens is removed, an artificial intraocular lens (IOL) is implanted, and the posterior capsule remains intact. (Bottom left) Over time, the posterior capsule may opacify, leading to blurred vision similar to a cataract. (Bottom right) YAG laser capsulotomy creates an opening in the cloudy posterior capsule, restoring the passage of light and improving vision.

Posterior capsular opacification is a common complication after cataract surgery, arising from residual lens epithelial cells proliferating on the capsular bag (1–3). This opacification of the posterior lens capsule causes light scattering and a decline in vision when it encroaches on the visual axis (4). While improved surgical techniques and intraocular lens (IOL) designs have reduced PCO incidence, it still affects up to 20% of patients within 5 years of cataract surgery (5). The standard treatment for visually significant PCO is neodymium: yttrium–aluminum–garnet (Nd:YAG) laser capsulotomy, which creates an opening in the opacified capsule (6). Nd:YAG capsulotomy is generally safe and effective, but it carries risks such as intraocular pressure spikes, IOL pitting or dislocation, cystoid macular edema, and retinal detachment (7). Decisions about if and when to perform a capsulotomy can be subjective, varying by patient symptoms, surgeon assessment, and resource availability (1, 7, 8). The clinical course of PCO and its standard management by Nd:YAG capsulotomy are illustrated in Figure 1.

**FIGURE 2:**
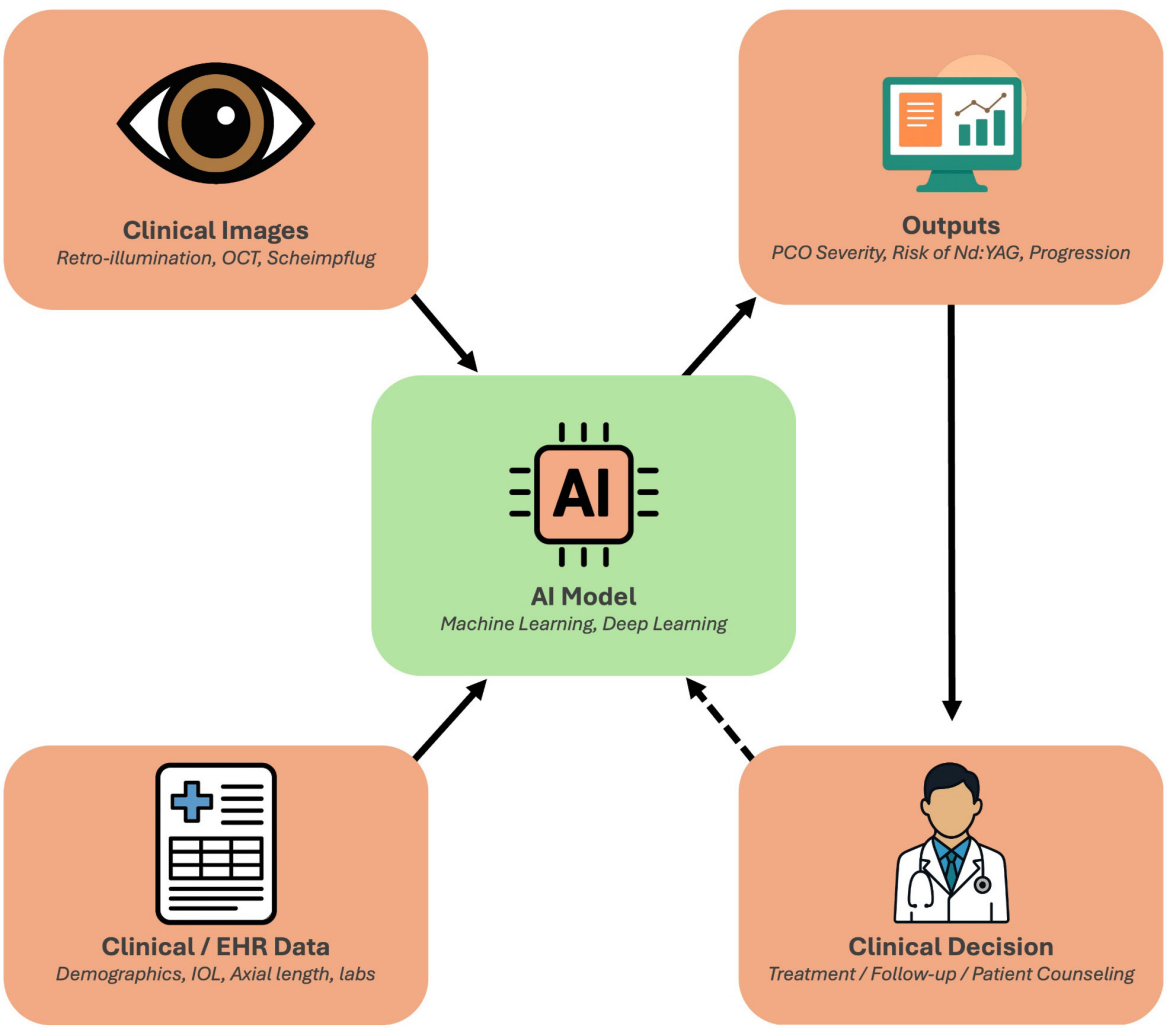
Conceptual framework of artificial intelligence (AI) in posterior capsule opacification (PCO) management. Clinical imaging data (retroillumination photographs, OCT, and Scheimpflug tomography) and clinical/EHR data (demographics, intraocular lens type, axial length, laboratory parameters) serve as inputs to AI models that integrate multimodal information through machine learning and deep learning algorithms. These models generate standardized outputs, such as PCO severity grading, progression estimates, and predicted risk of Nd:YAG capsulotomy, that support individualized clinical decisions regarding treatment timing, follow-up intervals, and patient counseling. The framework also incorporates a feedback loop, whereby real-world outcomes can continuously refine and improve model performance over time.

In the era of personalized medicine, there is a growing interest in leveraging AI to improve the care of PCO on an individual level (9). AI, encompassing machine learning (ML) and deep learning (DL) techniques, excels at analyzing complex data and could assist at multiple stages of PCO management, such as early diagnosis and objective grading of PCO, risk stratification for PCO development, decision support for timely intervention, and outcome prediction to tailor follow-up (1, 9). Notably, unlike retinal diseases, where AI screening is now well-established (e.g., diabetic retinopathy) (10–13), PCO and other anterior segment disorders have only recently seen AI applications (1). The potential is significant, as AI could standardize PCO evaluation (reducing observer bias), predict which patients are likely to develop significant preoperative and postoperative opacification, guide personalized timing of Nd:YAG treatment, and forecast patient-specific outcomes (1, 14, 15). This framework, linking multimodal inputs, AI analysis, and clinical decision making in PCO, is illustrated in Figure 2.

This article provides an in-depth review of AI-driven approaches in PCO management, organized by domain: (1) Diagnosis and Classification, (2) Risk Stratification and Prognostics, (3) Decision Support and Management, and (4) Outcome Prediction and Personalization. We highlight current capabilities in each domain, summarize key studies, and discuss how these advances enable more personalized PCO care. We also examine the challenges and limitations of AI in this field and outline future directions, including gaps such as the need for post-capsulotomy monitoring tools. The goal is to inform ophthalmologists and researchers of the state-of-the-art in AI for PCO and inspire further innovations in this field.

## 2 Methods

This review was conducted in accordance with the SANRA (Scale for the Assessment of Narrative Review Articles) guidelines to ensure methodological rigor, transparency, and balanced synthesis. A comprehensive literature search was performed across four databases, PubMed/MEDLINE, Scopus, Web of Science, and IEEE Xplore, covering publications from January 2010 through February 2025. Sentinel studies predating this period were included selectively when they provided foundational or contextual relevance, such as Koch (54) for Nd:YAG complications (6). Search terms combined condition-specific keywords (“posterior capsule opacification” OR “after-cataract”) with technology-related terms (“artificial intelligence” OR “machine learning” OR “deep learning”).

Studies were considered eligible if they (1) applied AI, ML, or DL methods to the detection, grading, risk prediction, or management of posterior capsule opacification, (2) analyzed human imaging or clinical data, and (3) were published in English. Excluded were animal and *in vitro* studies, narrative reviews or editorials without original analysis, and AI research in ophthalmology not directly addressing PCO. The included studies were synthesized qualitatively into five thematic domains: diagnosis and classification, risk stratification and prognosis, decision support and management, outcome prediction, and personalization.

In line with SANRA principles, we critically appraised the literature for study quality, identifying recurring limitations such as small sample sizes, retrospective single-center designs, limited external validation, and heterogeneity in imaging protocols. These constraints are explicitly acknowledged in the discussion to contextualize the strength and generalizability of current evidence. The goal of this approach is not exhaustive cataloging, but rather a structured and critical synthesis that highlights both the potential and limitations of AI in posterior capsule opacification management, while providing direction for future research.

## 3 AI applications in PCO

### 3.1 Diagnosis and classification of PCO

**FIGURE 3:**
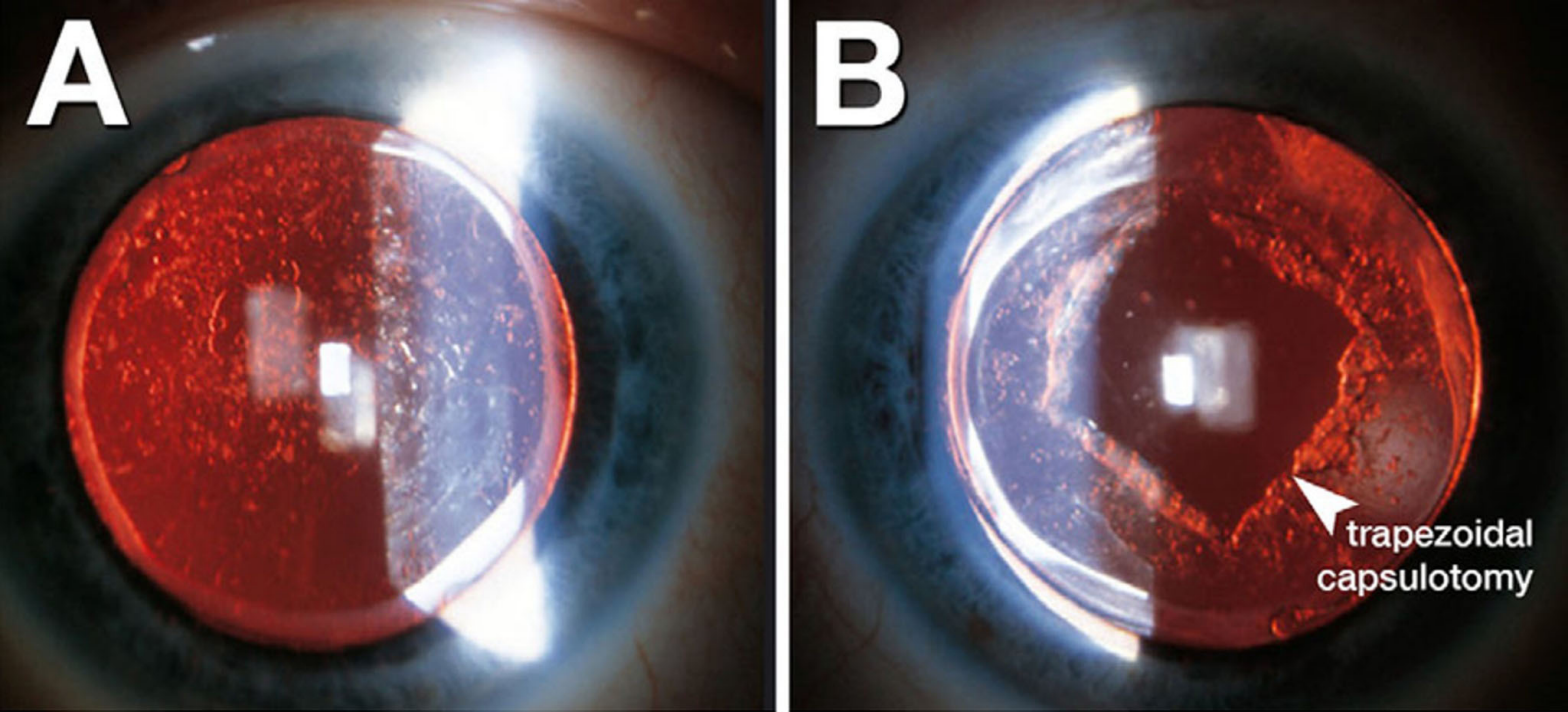
Representative grades of posterior capsule opacification (PCO). Slit-lamp retroillumination images illustrate the PCO grading scale: Grade 0, clear posterior capsule; Grade 1, wrinkling/opacity confined to a ∼4-mm central zone with clear view of the posterior pole; Grade 2, denser central/paracentral opacity that slightly obscures macular detail but not cup–disc assessment; Grade 3, opacity making the cup–disc ratio difficult to ascertain; Grade 4, dense central/paracentral opacity precluding adequate fundus view. *Reproduced, no changes made*, from Gu X, Chen X, Jin G, et al. Early-Onset Posterior Capsule Opacification: Incidence, Severity, and Risk Factors. *Ophthalmology and Therapy*. 2022;11:113–123. https://doi.org/10.1007/s40123-021-00408-4. © The Author(s) 2021. Licensed under Creative Commons Attribution-Non-Commercial 4.0 International (CC BY-NC 4.0); use permitted for non-commercial purposes with attribution and indication of changes. Link to license: http://creativecommons.org/licenses/by-nc/4.0/.

**FIGURE 4:**
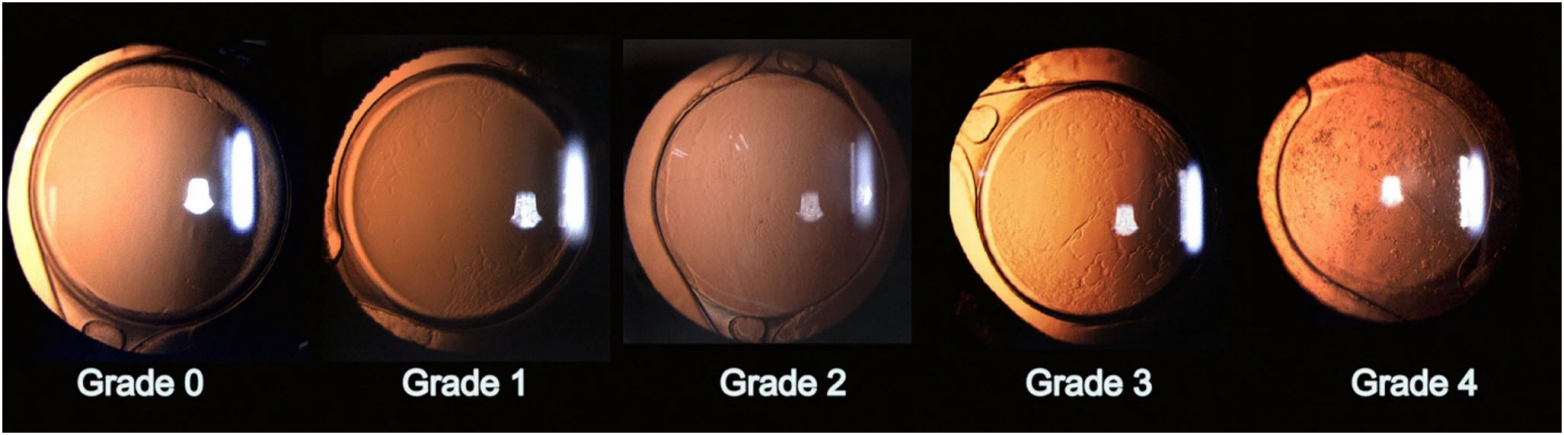
Retroillumination images of posterior capsule opacification (PCO). (A) Dense, diffuse PCO before Nd:YAG laser capsulotomy. (B) Post-laser view showing a trapezoidal capsulotomy opening through the opacified capsule. *Adapted (cropped) from* Berlin A, Clark ME, Swain TA, et al. Impact of the Aging Lens and Posterior Capsular Opacification on Quantitative Autofluorescence Imaging in Age-Related Macular Degeneration. *Transl Vis Sci Technol.* 2022;11(10):23. https://doi.org/10.1167/tvst.11.10.23. De-identified clinical images courtesy of Arno Sailer, MD. © The authors 2022. Licensed under CC BY 4.0; adapted with permission per license (https://creativecommons.org/licenses/by/4.0/).

Accurate detection and grading of PCO are critical for guiding timely intervention and optimizing patient outcomes. Historically, PCO severity has been evaluated via slit-lamp examination or subjective scoring systems applied to retroillumination images (1). Examples include the Evaluation of PCO (EPCO) and posterior capsule opacification (PCO) scoring software, which quantify the opacified area or density (1). The EPCO system grades PCO by dividing the posterior capsule into five concentric zones, each scored 0–4 for opacity density, and then multiplying each zone’s score by its relative area to produce a single continuous score reflecting both the extent and severity of opacification. Furthermore, the POCO system segments PCO on retroillumination images by having the operator manually set pixel intensity thresholds to isolate opacified regions, then calculates the percentage of the entire capsule area they occupy to yield a single area-based score. Although these manual methods provide useful estimates, they depend heavily on consistent imaging conditions and can introduce observer bias (1). To address these limitations, AI has been explored for the objective classification of PCO, thereby improving consistency and reproducibility. Representative slit-lamp retroillumination images showing the 0–4 grading spectrum of PCO are presented in Figure 3, and examples of dense diffuse PCO with post-laser clearance are shown in Figure 4.

Early AI-based strategies often employed classical image analysis, focusing on texture-based features such as the gray-level co-occurrence matrix (GLCM), a texture-analysis method that quantifies pixel intensity patterns in retroillumination images. For example, Liu et al. (55) introduced an enhanced GLCM-based model that computed texture metrics from retro-illumination photographs and utilized a support vector machine regressor to predict a continuous PCO severity score (16). By incorporating a regional “voting” mechanism centered on the visual axis, their algorithm’s severity outputs correlated strongly (*r* = 0.829) with expert ophthalmologists’ clinical grades, performing comparably to senior clinicians while outperforming less experienced graders (16). This underscores the potential of well-designed classical ML approaches to provide objective, expert-level grading. Other studies have similarly demonstrated that texture segmentation and co-occurrence metrics could mirror clinical assessments (16), lending additional support to the feasibility of quantitative imaging-based PCO evaluation.

Deep learning methods, particularly convolutional neural networks (CNNs), have further expanded the capabilities of AI-driven PCO classification. A notable example is the code-free deep learning system, an automated machine learning (AutoML) platform that enables clinicians to train CNNs without manual programming, as described by Huemer et al. (56). This system was trained on 279 retroillumination images graded from “no PCO” to “severe PCO” (17). The authors validated the model on an external set of 100 images, and it achieved a near-expert level area under the receiver operating characteristic curve (AUC) of 0.97 (95% CI, 0.93–0.99), with 84% sensitivity and 92% specificity in detecting clinically significant opacification (17). This high accuracy indicates that deep learning can reliably identify PCO cases that are likely to impair vision, potentially offering a standardized threshold for Nd:YAG capsulotomy. Clinically, eyes resembling Grades 2–4 in Figure 3 usually fall into the “treatment required” category, whereas Grades 0–1 are observed; similarly, Figure 4 illustrates the visual change before and after capsulotomy. Importantly, the system performed robustly even across different graders, suggesting it may alleviate observer variability and expand access to automated PCO evaluation for clinicians who are not programming specialists (17).

Some deep learning approaches have sought to grade PCO on a continuum or categorize it into morphological subtypes. For instance, the AQUA II system utilized texture analysis of retroillumination photographs to classify PCO into up to six distinct categories (1). Although not a deep network, AQUA II represented a reproducible automated classification solution that underscored how AI might capture the nuanced morphologic variations that inform clinical decision-making. Similarly, high-resolution OCT has also been harnessed to characterize capsular fibrosis and pearl deposits, thereby broadening the potential imaging modalities applicable to AI-based detection (1).

Building on classical machine-learning approaches, the AQUA II system applied texture analysis to high-resolution retroillumination photographs to classify PCO into six morphological subtypes with reproducible grades (1). Similar algorithms have been extended to OCT images to characterize fibrotic and pearl-type opacities (1). More recently, Peer et al. (57) developed an end-to-end convolutional neural network trained on 197 retroillumination images that automatically graded PCO severity across three-class and binary schemes as well as AQUA-style cutouts (18). When borderline Class 1 cases were excluded, the CNN achieved 90% accuracy, surpassing AQUA II’s best of 86%, and required substantially less manual preprocessing. Even with AQUA-style cutouts, the model achieved 67% accuracy (versus 62% for AQUA II), and Grad-CAM heatmaps confirmed focused attention on clinically relevant opacity regions, although borderline cases remain challenging. These findings demonstrate that deep learning architectures can deliver expert-level performance on modest datasets, highlighting their translational promise for resource-limited or real-time clinical settings. Altogether, AI-driven image analysis, whether via classical texture-based ML or advanced CNN frameworks, offers objective, reproducible PCO grading with diagnostic accuracies that rival seasoned ophthalmologists (1), potentially enabling earlier detection of vision-threatening opacification and reducing both under- and overtreatment (Table 1). This spectrum of severity (Figure 3) and its resolution with capsulotomy (Figure 4) are the very phenotypes that AI algorithms are trained to recognize and quantify.

**TABLE 1:** Summary of AI-driven models for posterior capsule opacification detection, classification, and prognostication.

Study	AI method/ architecture	Input variables	Image/data modality	Predicted output	Dataset size and split	Reported performance	Critical notes and validation details
Mohammadi (58)	Feed-forward ANN	Demographics + ocular history	Clinical tabular data	Clinically-significant PCO at 2 years	352 eyes	AUC 0.71; Sp 97%, Sn 25%	Proof-of-concept; high specificity, low sensitivity
Jiang (TempSeq-Net) (84)	CNN + LSTM sequence model	Slit-lamp images/eye	Color slit-lamp photos	Future Nd: YAG needs	6 090 images (1 015 pts)	Acc 92.2%, AUC 97.18%	Real-time progression predictor
Chang (59)	Logistic-regression nomogram	Age, surgery type, intraocular lens (IO)L, etc.	EHR variables	Nd: YAG benefit risk	312 pts (7:3 split)	C-index 0.870/0.842	Decision-curve analysis included
Abràmoff (85)	CNN (DR detection), no PCO	Fundus + OCT images	Retinal imaging	DR severity	819 pts	Sn 87%, Sp 91%	Listed only to note the absence of PCO model
Ghamsarian (86)	R-CNN + U-Net + RNN	Surgical video frames	Microscope videos	Lens rotation/instability	Large video set (NNR)	Mean error 3.7°	Not PCO-specific
Huemer (56)	AutoML Vision CNN	Retro-illum. pixels	High-res photos	Clinically-significant PCO	179 train/100 test	AUC 0.97 external; Sn 0.84 Sp 0.92	Code-free pipeline
Li (28)	Cox + LASSO nomogram	Age, sex, IOL, myopia, fibrinogen	EMR + labs	1-, 3-,5-years Nd: YAG risk	9,768 eyes (70/30)	AUC ≈ 0.69 (5 years)	Hydrophobic IOL benefit lost in high-myopes
Hatamnejad (87)	AI voice-assistant protocol	Telephone dialogue	Voice	Safety/usability	Planned 500 pts		Post-cataract triage study
Lu (88)	ROC thresholding (no ML)	Objective Scattering Index (OSI)	Double-pass OQAS images	Benefits of Nd: YAG in mild PCO	105 eyes	OSI AUC 0.996 (VA > 0.1 logMAR)	OSI outperforms CDVA for referral decision
Liu (55)	GLCM + SVM (three variants)	Texture features	Retro-illum. photos	Continuous PCO severity score	100 pts	Best model *r* = 0.829 vs. experts	GLCM + V beats junior clinicians; no bias
Shrestha (89)	U-Net segmentation → rule-based classifier	Retro-illum. images	Retro-illum. photos	“Treatment required” vs. “not yet”	(N not stated)	Dice > 0.80; F2-score 0.98	Automated GT performs as well as manual
Ursell (90)	Real-world cohort stats	EMR data	20,763 eyes	5-years Nd: Y AG and PCO incidence	Inc 5.8–19.3% (Nd: YAG)	Hydrophobic acrylic has the lowest risk	
Meacock (91)	Texture threshold study	% central PCO	Retro-illum. photos	Impact on VA, CS, scatter	106 eyes	≥78% PCO impacts high-contrast VA; light-scatter sensitive at <1%	Links quantitative PCO to psychophysics
Vasan (92)	Smartphone CNN cataract screener (e-Paarvai)	Rear-camera eye photos	Smartphone images	4-class cataract grading	2,619 eyes	Cataract detect Se 96%, Sp 25%; overall acc 88%	High PPV for immature cataract; lower for mature and PCIOL

This table compiles key details from primary studies applying machine-learning and deep-learning methods to PCO, including the AI architecture used, types of input data (clinical, imaging, electronic health record, or surgical video), predicted clinical outputs (e.g., 2-years PCO occurrence, need for Nd: YAG capsulotomy, severity grading), dataset size and train/test splits, core performance metrics (AUC, sensitivity, specificity), and critical validation notes or limitations (single-center vs. multicenter cohorts, internal vs. external validation). Together, these entries illustrate how diverse inputs, from demographics and slit-lamp photographs to intraoperative metrics, have been leveraged to create proof-of-concept pipelines and risk-stratification tools, while also highlighting the need for broader external testing and workflow integration prior to clinical deployment.

Conventional systems, such as EPCO and POCO, remain the reference standard in many clinics; however, they are time-consuming and vulnerable to inter-observer variation and image-capture inconsistencies. Recent AI approaches (e.g., GLCM-SVM and CNNs) reproduce expert-level severity estimates while minimizing operator dependency and preprocessing. In external testing, they can match or exceed clinician performance in detecting clinically significant PCO. A pragmatic path forward is dual reporting, an AI-derived continuous severity index displayed alongside EPCO/POCO values, so that surgeons can validate automated outputs against familiar scales during a transition period. This alignment facilitates adoption, allows local calibration, and enables service-level audits of agreement (e.g., correlation with EPCO, Bland–Altman limits, and κ for “treat vs. observe”). Studies such as Huemer (AutoML, AUC ≈ 0.97, 84%/92% external Se/Sp) and Liu (r ≈ 0.83 vs. experts) suggest that AI scores around the upper tertile often coincide with clinician decisions to treat, whereas lower scores map to observation (17). Coupling the AI index with objective scatter from double-pass systems (e.g., OSI) provides convergent evidence: Lu et al. showed OSI-based thresholds strongly track function and referral decisions, outperforming CDVA alone. An AI + OSI composite could stabilize decisions across lighting and imaging variability and standardize capsulotomy referral criteria across sites.

Artificial intelligence-driven image analysis has the potential to enhance the objectivity and reproducibility of PCO diagnosis, whether through classical texture-based ML methods or advanced deep learning frameworks. Recent studies report diagnostic accuracies and correlation metrics comparable to those of seasoned ophthalmologists, suggesting that integrating these tools into clinical workflows could flag vision-threatening PCO earlier and reduce the chance of undertreatment or overtreatment, as shown in Table 1. Moreover, automated and quantifiable grading may provide a valuable baseline for tracking PCO progression over time and ensure that capsulotomy decisions are based on consistent, data-driven criteria.

### 3.2 Risk stratification and prognostics

The clinical trajectory of PCO is highly heterogeneous: some patients harbor mild, asymptomatic opacities for years, whereas others develop rapid, vision-threatening fibrotic changes within months. This variability complicates postoperative planning, patient counseling, and resource allocation. Recognizing these challenges, investigators have begun to leverage machine-learning prognostic models.

For instance, Mohammadi et al. (58) developed one of the first artificial neural networks for PCO prognosis, training on ten perioperative variables from 352 eyes to forecast 2-years clinically significant opacification with 87% accuracy (specificity 97%, sensitivity 25%), thereby validating known risk factors such as younger age and certain IOL designs (14). While the imbalanced performance meant that some actual PCO cases were missed, the model validated known risk factors, such as younger age and certain IOL haptic angulation or edge features, and demonstrated the feasibility of ML for PCO prediction at a time when AI in ophthalmology was still nascent. Subsequent investigations have utilized larger datasets and more sophisticated deep learning architectures. Jiang et al., for example, introduced a temporal sequence network (TempSeq-Net) that combined CNNs for feature extraction with a long short-term memory (LSTM) module to capture disease progression across consecutive slit-lamp photographs (15). Their approach, trained on more than 6,000 images, predicted whether mild PCO would advance to a visually significant stage requiring Nd:YAG laser capsulotomy with an area under the receiver operating characteristic curve (AUC) of around 0.97, accompanied by high sensitivity (>92%) and specificity (∼81%) (9, 15). Operationally, this corresponds to the progression depicted in Figure 1, from post-cataract clarity to capsule opacification and, when indicated, laser capsulotomy. This time-series design highlights the advantage of incorporating longitudinal imaging data, enabling a more dynamic prognosis that can alert clinicians to early capsular changes well before they become symptomatic.

Beyond imaging, modern cataract registries now capture not only postoperative images but also extensive preoperative and intraoperative datasets, including patient demographics, biometric measurements, surgical parameters, and even biochemical markers, that can be leveraged by AI-driven algorithms for more nuanced risk stratification. Machine-learning models can be applied to these registry data to highlight the predictive importance of factors such as IOL material (hydrophilic versus hydrophobic), axial length, surgical technique, and select biochemical biomarkers in determining PCO severity (19). In a large retrospective cohort of 9,768 eyes, Li et al. identified younger age, male sex, and hydrophilic IOL material as key predictors of early laser capsulotomy. The nomogram achieved time-dependent AUCs of 0.650, 0.683, and 0.678 for predicting 1-, 3-, and 5-years Nd:YAG capsulotomy risks in the training cohort. In their internal validation split (*n* = 2,930), these rose modestly to 0.702, 0.691, and 0.688, respectively, suggesting minimal overfitting despite reliance on clinical variables alone. No true external validation on an independent dataset was reported; therefore, the model’s generalizability beyond the original registry remains untested (19). Chang et al. further explored a nomogram-based model for PCO prognostication in 312 patients and reported a C-index of 0.87 in internal validation; however, the external applicability remains to be confirmed (20). These predictions correspond to the “Outputs” component shown in Figure 2, where risk of Nd:YAG is one of the model’s key deliverables. Collectively, these findings highlight that comprehensive, multi-factor models can predict who is at highest risk for the development of PCO, including younger, highly myopic patients or those receiving certain IOL designs.

Prognostic models (e.g., Mohammadi ANN; Jiang TempSeq-Net; Li/Chang nomograms) can do more than label risk; they can assign follow-up intensity. For example, a “high-risk” output (such as younger age, hydrophilic IOL, long axial length, or early texture changes) could trigger a 3-months review with imaging, whereas a “low-risk” output could safely extend to 12 months and be monitored with tele-triage or smartphone capture, where feasible. While specific intervals require prospective validation, the manuscript now clarifies that risk-tiered schedules are the logical clinical expression of AI predictions and are testable in pragmatic trials (outcomes: time-to-capsulotomy, VA/contrast, patient-reported glare, and visits avoided). Registry-based data show YAG rates and complication profiles vary by age, axial length, and material. Integrating these priors with AI risk helps identify eyes where clinical observation is safer (e.g., low risk + low OSI + pericentral opacity) versus those where earlier YAG is justified (high risk + central opacity + rising OSI). This reframes management as a risk-harm trade-off rather than a single threshold, and the manuscript now provides explicit examples of how to operationalize this logic in the clinic.

While most approaches rely on either static clinical or imaging data, emerging research suggests that real-time or intraoperative metrics could also inform PCO risk. For instance, analyzing surgical videos for incomplete lens cortex removal or inadequate capsulorhexis overlap may offer predictive signals, mirroring work in other domains where CNNs applied to operative footage help forecast postoperative complications (9, 21). Although studies in this vein are still sparse, they suggest a future in which AI systems provide immediate feedback during cataract surgery itself, potentially reducing the incidence of PCO by prompting more thorough capsule polishing or more careful IOL positioning.

Artificial intelligence can also predict long-term, patient-specific outcomes after Nd:YAG capsulotomy, most notably the likely visual gain each patient is expected to achieve. Although no dedicated AI model exists yet, combining detailed optical metrics, imaging features, and comorbidity profiles has already shown promise for forecasting post-laser improvement, even in eyes with coexisting pathologies, such as macular degeneration (22). Equally valuable is identifying patients at elevated risk for post-YAG complications such as residual anterior capsule opacification, edge contraction in pediatric cases, retinal detachment, or cystoid macular edema. An extensive registry study quantified these risks, finding that younger, highly myopic eyes are particularly vulnerable (23). While that analysis wasn’t AI-based, it highlights how axial length, timing, and other factors can feed predictive algorithms. A future AI tool could flag high-risk patients and recommend extra retinal follow-up or precautionary measures. Although no peer-reviewed AI model for post-YAG forecasting currently exists, current work on PCO risk stratification (19, 20) and large-scale registry data (23) lays the groundwork. Personalization extends to prevention. If a preoperative AI model [Li et al. (28); Chang et al. (59)] flags a patient, say, a young uveitic eye or high myope, as high-risk for PCO, the surgeon can choose sharp-edged hydrophobic acrylic IOLs, meticulous capsular polishing, or even a primary posterior capsulotomy in pediatric cases to lower incidence. Although current practice relies on a consensus regarding risk factors, future AI could integrate uveal inflammation status, axial length, and genetic data into a personalized surgical plan. Early nomogram studies (19, 20) already predict Nd:YAG likelihood and highlight modifiable factors, such as IOL type, at extraction. A related frontier is forecasting patient-centric outcomes, including the quality of life and satisfaction. An AI could learn that someone with high visual demands (e.g., a pilot or microsurgeon) suffers more from mild PCO than a less visually stressed individual. While current models focus on acuity or contrast, integrating patient-reported outcomes (PROs) (8) would enable algorithms to advise earlier capsulotomy for those who require crisp vision and more extended observation for others. This patient-first strategy embodies personalized medicine but awaits the availability of larger datasets and PRO integration.

Post-capsulotomy care offers another AI opportunity. After PCO clears, standard cataract follow-up resumes; however, capsulotomy can trigger IOP spikes, retinal tears, or floaters (7, 23). An AI that stratifies post-YAG risk could schedule closer retinal checks for young, highly myopic patients and shift low-risk individuals to telemedicine or AI-led phone triage. Emerging systems (24) use structured calls or apps to flag red-flag symptoms and prompt office visits only when needed, enhancing safety and reducing unnecessary follow-ups.

Collectively, these AI-driven efforts have reframed PCO risk stratification as a proactive, patient-specific process. By utilizing complex inputs, demographic risk factors, imaging biomarkers, and procedural details, patients can be grouped into risk tiers for visually significant opacification (19). This segmentation enables ophthalmologists to individualize their follow-up protocols, intervening early for those likely to progress and sparing unnecessary clinic visits for low-risk individuals (9). Although several challenges remain, notably model generalizability, prospective validation, and streamlined workflow integration, the accumulating evidence suggests that AI-based prognostic tools may aid in managing PCO across a diverse patient population.

Outcome prediction and personalization mark the next frontier in AI-enhanced PCO management. Beyond pinpointing who will develop PCO and when to perform capsulotomy, future models will estimate each patient’s visual gain from intervention, forecast potential complications, and tailor follow-up intensity accordingly. Paired with advanced AI-based detection and risk stratification, these individualized strategies promise a future where every patient’s care is precisely calibrated. By leveraging real-time analytics on large clinical datasets, AI will drive customized surgical planning, optimal treatment timing, and personalized post-laser monitoring. Moving past a binary “treat or observe” paradigm, these tools aim to optimize long-term visual outcomes and elevate PCO care to a truly patient-centric standard.

### 3.3 Decision support and management

Artificial intelligence-based decision support tools aim to assist clinicians in determining when and how to manage PCO for optimal patient outcomes. One of the most pressing management questions is timing: waiting too long to perform Nd:YAG capsulotomy risks prolonged visual impairment, whereas intervening too early introduces unnecessary procedural risks and healthcare costs. In practice, the decision to proceed with laser capsulotomy typically hinges on subjective factors such as visual symptoms (e.g., decreased acuity, glare) and the ophthalmologist’s clinical judgment regarding the severity of PCO. Conceptually, this process is the right-hand arc of Figure 2, where model outputs inform clinical decisions. An AI-driven framework may transform this process into a more data-driven and standardized approach by objectively assessing the significance of opacification and, in some cases, suggesting an ideal intervention window.

A practical decision framework can be derived by integrating AI-derived severity indices with complementary metrics such as the Objective Scatter Index (OSI). In this framework, patients with high AI-derived severity scores, coupled with elevated OSI values and central involvement, are classified as candidates for immediate treatment. Those with moderate severity or borderline OSI values may be allocated to short-interval follow-up (e.g., 1–3 months), while eyes with low AI scores and stable OSI trajectories may be safely scheduled for deferred review (6–12 months). This tiered approach provides a structured means of translating algorithmic outputs into actionable clinical decisions while accommodating patient-reported symptoms and functional demands.

A practical illustration of AI-enabled decision support is the binary classification of PCO cases into “treatment required” versus “observation” (17). In a proof-of-concept study, Huemer et al. (56) leveraged Google AutoML’s “no-code” interface to train a CNN on 179 retro-illumination images, 90 labeled as “treatment required” (clinically significant PCO) and 89 as “observation,” using expert Nd:YAG capsulotomy decisions as the ground truth (17). After optimizing model hyperparameters automatically within the platform, they internally validated on a held-out set (20% of the dataset), achieving 94% sensitivity and 97% specificity for detecting treatment-worthy opacities. When tested on an independent external cohort of 100 images, the CNN maintained strong performance, with 84% sensitivity and 92% specificity (17). This type of tool could be readily integrated into routine exams: a postoperative photograph is analyzed by the AI, which reports a severity index (e.g., “PCO severity: 0.85 [high] → likely requires YAG laser”). In real-world deployment, it could help standardize indications for Nd:YAG by ensuring that each patient is evaluated with consistent criteria, thereby mitigating variability caused by clinician expertise or patient preference (1). Other teams have demonstrated similar workflows. For example, Shrestha et al. trained a U-Net CNN to segment the opacified area on slit-lamp images and classify cases as “treatment required” or “not yet required,” suggesting that follow-up intervals could be tailored to an individual’s PCO progression risk rather than uniform scheduling (25). Wu et al. likewise incorporated PCO assessment into a broader AI platform for comprehensive cataract management, ensuring that cases requiring timely laser treatment were identified, even in remote or primary care settings (26).

Researchers have also investigated AI-guided methods to reduce the burden on both patients and clinics by personalizing follow-up plans. One proposed mechanism is to use automated classification to triage patients who require close in-person follow-up versus those whose mild PCO can be safely monitored at longer intervals or via telemedicine. This resonates with Shrestha et al.’s work, where the U-Net-based segmentation approach quantitatively estimates the opacified area (25). When that metric surpasses a predefined threshold, the AI flags the case for earlier intervention. Conversely, a patient whose PCO is well below the threshold could be seen less frequently, thus reducing unnecessary clinic visits. Beyond scheduling optimization, AI can support more targeted treatment protocols. Suppose the automated analysis reveals dense or centrally located opacification. In that case, it might inform the surgeon’s choice of laser energy or capsulotomy pattern, although published data specifically guiding these parameters remain limited or unexplored.

Another benefit is the AI’s potential to discriminate PCO from other causes of vision decline, such as macular pathology. Systems capable of analyzing both anterior and posterior segment imaging (e.g., slit-lamp photographs and OCT scans) could confirm whether decreased vision primarily stems from the opacified capsule rather than co-existing diseases, thereby preventing an unnecessary capsulotomy (26). Wu et al. demonstrated this in a three-stage, cascaded CNN pipeline trained on a multi-center dataset of slit-lamp photographs and anterior-segment OCT images. In the first stage, a ResNet-based classifier distinguished imaging modes (slit-lamp vs. OCT vs. external photograph) with 98% accuracy. The second stage employed a U-Net architecture to segment the lens and capsular region, achieving a mean Dice coefficient of 0.88 for capsule delineation. Finally, a custom Inception-based CNN ingested the segmented regions to assign each eye to one of four postoperative states (normal, residual/recurrent cataract, PCO, or other anterior pathology), yielding an overall accuracy of 91% and an AUC of 0.92 for PCO detection on an independent test set (26). This type of multimodal integration reflects the central pathway in Figure 2, combining imaging and EHR inputs to guide management.

Despite these advances, it remains crucial to note that current AI tools should complement, not supplant, the ophthalmologist’s clinical judgment. Risk estimates or severity scores generated by an algorithm may occasionally differ from a physician’s assessment. A missed indication for capsulotomy (false negative) could delay essential care, whereas a false alarm risks unnecessary procedures. Encouragingly, the high-specificity design of most PCO AI models (17) lowers the likelihood of recommending YAG where it is not warranted. Moreover, as AI systems gather real-world data across diverse populations and imaging conditions, it is hoped that performance will be continuously refined, further reducing error rates. Maintaining patient safety and ethical oversight is therefore paramount when integrating AI recommendations into routine practice.

Artificial intelligence-based decision support in PCO management shows promise for improving the precision and consistency of Nd:YAG laser timing, individualizing follow-up intervals, and accurately identifying eyes that truly require treatment. By reducing reliance on subjective impressions, these systems can minimize both undertreatment and overtreatment, promoting better resource allocation and safeguarding long-term visual outcomes.

## 4 Challenges, limitations, and future directions

While the promise of AI in PCO management is evident, several challenges must be addressed before these tools can be widely adopted in clinical practice. These issues range from the technical limitations of current models and the need for diverse, high-quality datasets to challenges in regulatory approval, workflow integration, and clinician trust. The following sections outline these obstacles, suggesting possible steps needed to incorporate AI innovation into ophthalmic practice.

A robust AI model requires large, high-quality datasets that represent the diversity of real-world patients and imaging conditions. In PCO, obtaining such data is challenging, and data heterogeneity (e.g., variations in image focus, pupil dilation, or co-existing ocular conditions) can decrease the accuracy of AI models. Many published AI models designed to evaluate PCO are trained on a few hundred images or cases, often from a single center (16, 17). This raises concerns about overfitting and limited generalizability as clinics may use different cameras, lighting, or have patient populations with various risk profiles. An algorithm trained on one type of slit-lamp photography might perform less well on another. Moreover, annotated PCO datasets are labor-intensive to create as they require expert grading of thousands of images. Efforts to build larger multi-center databases, possibly through federated learning collaborations, where models are trained across sites without sharing raw images, will be needed to improve model robustness. However, AI systems rely on patient data, which raises privacy issues. Imaging data and health records used for model training must be handled in compliance with privacy regulations specific to each country or governing organization. If data from multiple centers or countries is combined, navigating different privacy laws becomes complex (27). Federated learning has already shown promise in ophthalmology, particularly when evaluating retinal imaging, enhancing generalizability in diabetic retinopathy, and improving OCT segmentation while preserving privacy (28, 29).

Demonstrating clinically acceptable performance of AI systems used in the management of PCO is important to consider when contemplating their role in ophthalmology. High accuracy or AUC in a paper does not always translate to consistent performance in live clinical settings. Models must be prospectively validated on new patients and ideally compared against the standard of care in outcome studies. For example, does using the AI to decide on YAG timing improve visual outcomes or patient satisfaction compared to the current standard? At the time of this review, most studies report retrospective performance. Moving to prospective clinical trials or at least real-time testing is a crucial next step, as regulatory approval will hinge on evidence that the AI is both safe and effective. This is particularly important if an AI tool is to influence a treatment decision directly.

Many AI models, especially deep learning CNNs, operate as black boxes, providing a result without an easy explanation (55). In medicine, this opacity can hinder trust and adoption. Clinicians may be reluctant to act on a recommendation that they cannot interpret or justify to a patient. Explainable AI approaches, such as saliency maps or feature attribution methods, can bridge this gap by linking model outputs to clinically meaningful features, thereby enhancing transparency and interpretability. For example, highlighting which regions of the capsule influenced a prediction may help clinicians justify treatment decisions. Combining interpretable features, such as quantifiable PCO area and density, with deep learning methods offers one strategy to address these concerns. Some current systems do provide partial explanations (like showing the segmented opacified area), but more transparent decision logic will increase clinician confidence.

Beyond technical limitations, the clinical deployment of AI in PCO management raises regulatory and ethical challenges. In the United States, FDA approval is required for the clinical implementation of AI algorithms, typically under Software as a Medical Device (SaMD) frameworks. In Europe, CE marking serves as a parallel certification. Regulatory pathways emphasize not only accuracy but also reproducibility, transparency, and clinical validation. A particular challenge for AI in ophthalmology is the adaptive nature of some algorithms, which may require continuous oversight to ensure safety after deployment. Liability is another important consideration: if an AI system recommends delaying Nd:YAG capsulotomy and the patient suffers visual harm, responsibility may be unclear between the developer, the institution, and the treating physician. Clear legal frameworks and shared accountability models are needed. Finally, informed consent will be critical for adoption. Patients should be explicitly informed when AI contributes to their clinical evaluation, including the benefits, limitations, and interpretability of the algorithm. Transparent communication, combined with clinician oversight, will help maintain trust and safeguard ethical standards as these tools move toward clinical use.

Introducing AI into the clinical workflow presents logistical challenges. Clinics would need the capability to capture standardized images of the posterior capsule, possibly at each visit, and then run the AI analysis quickly, as it would likely be done in real-time during the patient exam. This might require new hardware or software integration into existing electronic medical record systems or imaging devices. There may be a learning curve for technicians to acquire images of sufficient quality for the AI to analyze. Additionally, if an AI flags a case for earlier YAG, there must be a clear pathway for that information to be translated into action (e.g., scheduling the laser procedure). Looking ahead, integrating AI-powered clinical decision support systems that can process slit-lamp photos or videos to provide real-time alerts, similar to intraoperative “smart instruments” used in cataract surgery (30, 31). These systems would need to be interoperable with EMRs and imaging devices through international data standards like Digital Imaging and Communications in Medicine (DICOM) and Health Level Seven International (HL7) to enable image capture, automated analysis, and action-triggered workflows. Workflow integration has been a barrier in other AI applications, and user-friendly implementations will be key for PCO as well.

Future models may combine multiple data sources to adopt a multi-modal approach and provide a more comprehensive assessment as compared to a unimodal approach. More recent work by Zhou et al. (60) conducted a cross-sectional analysis of 162 post-phacoemulsification eyes, including 65 eyes requiring capsulotomy and 97 controls, using swept-source OCT to measure posterior capsule thickness (PCT) and Pentacam Scheimpflug imaging for gray-value densitometry (32). Eyes needing Nd:YAG had significantly higher mean PCT (8.0 ± 2.7 vs. 5.0 ± 0.9 pixels) and gray values (66 ± 33 vs. 11 ± 17 pixels), with ROC AUCs of 0.942 (85% sensitivity, 74% specificity) for PCT and 0.947 (91% sensitivity, 76% specificity) for gray-value measures. These findings provide a concrete precedent for integrating tomographic thickness measures with Scheimpflug densitometry, suggesting that incorporating slit-lamp retroillumination data into a truly multimodal AI framework could potentially further enhance the accuracy and early detection of vision-threatening PCO (14, 32). In terms of workflow, this aligns with the multimodal input streams depicted in Figure 2. An AI system that inputs all three could compensate for the weaknesses of any single modality and give a more holistic evaluation of PCO. Early attempts in this direction (e.g., AQUA II, which combines retro images and Pentacam) show promise (14), but a deep learning model that fuses modalities might achieve even better performance. This could also extend to incorporating pre- and post-operative biometry and vision data to predict the simultaneous impact on visual function.

With the rise of telemedicine and patient engagement, future tools might enable patients to participate in monitoring their PCO status. For instance, a smartphone application could be developed where patients take a standardized photograph of their eye (with the flash producing a red reflex) at intervals, and an embedded AI model evaluates the PCO. While currently smartphone AI apps have been directed at cataract or refractive checks (33), extending this to PCO could enable low-resource or remote follow-up. A validated home-monitoring AI app could alert patients to see their ophthalmologist when PCO worsens beyond a threshold, thus personalizing follow-up frequency to actual disease progression rather than fixed schedules. Such tools would effectively add a new input channel, completing the feedback loop in Figure 2.

Beyond PCO, recent ophthalmic surgery studies have leveraged AI for risk stratification, outcome prediction, and intraoperative decision support. In glaucoma surgery, multimodal models that combine structured EHR with operative notes improved multiclass outcome prediction after trabeculectomy (macro-AUROC 0.75) (61); image-based deep learning on slit-lamp photos classified filtration bleb function with AUROC 0.80 in external testing (62), and machine-learning models predicted failure after Ahmed valve implantation at 1 year with AUROC up to 0.80 when systemic and medication data were added (63). For rhegmatogenous retinal detachment (RRD), multimodal fusion of preoperative OCT and ultra-widefield imaging predicted 3-months visual impairment with an AUROC of 0.91 (64), and a separate deep learning system predicted anatomical success with an AUROC of 0.94 (65). In corneal endothelial keratoplasty, algorithms using intraoperative OCT automatically assessed DMEK graft orientation to support intraoperative decisions (66). Meanwhile, preoperative AS-OCT–based deep learning predicted the risk of postoperative graft detachment (rebubbling), enabling targeted perioperative planning (67). In cataract surgery, the dominant AI focus has been (i) refractive planning using ML/DL IOL-power formulas–which consistently reduce mean absolute error versus conventional vergence methods in contemporary series and reviews–and (ii) surgical video analytics for phase recognition and automated performance feedback; together these tools aim to prevent complications such as posterior capsular rupture (PCR) and standardize training (68–70). Taken together, outcome-prediction AUROCs reported across non-PCO surgical tasks typically fall in the ∼0.74–0.94 range depending on the endpoint, data sources, and modeling strategy (61–64). This literature supports the clinical premise of our work–i.e., that supervised models can learn actionable peri- and postoperative signals in ophthalmic surgery–and provides performance benchmarks that help contextualize our findings within a broader, rapidly maturing evidence base (61–67).

To improve adoption, future AI systems may include interactive features that allow clinicians to adjust sensitivity or thresholds. For example, an AI might enable the ophthalmologist to set what they consider “significant PCO,” and the model output adjusts accordingly. This kind of user-tunable AI would make the system more flexible and align with personalized thresholds of different practitioners (71–74). In addition, integrating explanations, like showing which part of the capsule was most opacified, can help standardize grading and subsequent laser intervention. To further facilitate the adoption of AI, future directions must also include rigorous trials to demonstrate the benefits of AI-driven PCO care. These studies could compare standard follow-up schedules with AI-guided schedules, or immediate YAG with AI-suggested timing, measuring outcomes such as vision, patient satisfaction, complication rates, and cost-effectiveness. If AI can safely reduce the number of unnecessary follow-up visits or delay surgeries without affecting vision outcomes, the cost savings and efficiency gains should also be quantified. Conversely, if earlier intervention guided by AI leads to a better quality of life, that should be evidenced. Health economic analyses will be crucial for healthcare systems to adopt these technologies effectively.

An often-cited challenge is gaining the trust of end-users, who are generally ophthalmologists and patients. Clinicians need to feel confident that the AI will help rather than hinder their practice (75–79). Education and familiarity with these technologies can help overcome the hesitation that may be expressed by individuals who are wary of AI developments (80). Early adopters and clinical champions can then demonstrate the benefits. Some studies have been conducted using “code-free” platforms involving ophthalmologists in model training, which can also be done. This may improve trust in these systems, as clinicians become more involved in the AI development process (17). Regardless of the clinician’s role in the AI development and deployment, patients should be informed when AI is used in their care. Some patients may be wary of an AI influencing their treatment, so transparency and assurance that AI complements, not replaces, the physician’s expertise are essential for acceptance (80).

Furthermore, gaps still remain underexplored in the current literature. First, most AI systems for PCO have been benchmarked against surrogate measures such as EPCO or POCO scores, which are prone to subjectivity and do not necessarily reflect functional vision. Few studies have incorporated outcomes that matter most to patients, such as visual acuity, contrast sensitivity, glare disability, or patient-reported quality of life (81–83). Prospective trials should integrate these functional endpoints alongside algorithmic outputs to ensure that AI-driven recommendations translate into tangible visual benefits.

Second, population diversity remains limited. Many models are developed from single geographic or ethnic cohorts, raising questions about generalizability to broader patient populations. Ensuring performance across varied demographic and clinical contexts will require large, multicenter datasets and systematic cross-validation across devices and institutions.

Third, cost-effectiveness and system-level impact have yet to be rigorously studied. While AI tools are often promoted for their efficiency, formal health economic evaluations–including budget impact, cost per quality-adjusted life year, and decision-curve analysis–are needed to quantify their value in real-world practice. Such analyses should assess not only improved accuracy but also reductions in unnecessary Nd:YAG procedures, optimized follow-up intervals, and patient satisfaction.

Finally, future research should adopt a structured roadmap: multicenter prospective registries with standardized imaging protocols, trials directly comparing AI-assisted decision-making against conventional care, and evaluations of outcomes such as unnecessary capsulotomy avoidance, complication rates, and economic efficiency. By addressing these evidence gaps, AI in PCO management can move from proof-of-concept toward safe, equitable, and cost-effective clinical integration.

## 5 Conclusion

Posterior capsule opacification remains a key postoperative challenge in cataract surgery. AI-driven tools can streamline every step, from detecting and grading opacities to stratifying risk, optimizing intervention timing, and forecasting outcomes, by converting subjective assessments into objective metrics such as area, density, and progression risk. By mining large datasets, these systems reveal subtle predictors clinicians might miss, enabling truly personalized care plans. Modern AI systems can match expert performance in identifying clinically significant PCO, accurately forecast its progression, and eliminate unnecessary delays or procedures. This precision enables low-risk patients to skip intensive follow-up, while directing timely treatment to high-risk eyes, thereby maximizing clinic efficiency and avoiding low-value interventions.

Implementing these advances demands rigorous validation, seamless integration into clinical workflows, and strong clinician buy-in. Just as AI in retinal screening overcame early hesitation to become routine, applying these tools to anterior segment care is the next logical step. Crucially, AI must augment, not replace, the ophthalmologist, acting as an always-available, data-driven second opinion.

Clinicians also need to remain aware of AI’s limitations and combine machine consistency with human judgment. When AI and physician recommendations diverge, for example, AI flags a need for YAG but the surgeon decides to observe, it should trigger a careful review of both the model’s data and the patient’s unique context, ensuring technology informs rather than dictates care.

Artificial intelligence is transforming PCO care by enabling more intelligent detection and intervention, so each patient receives the proper treatment at the right time. Through interdisciplinary collaboration and rigorous research, we’re on track to manage PCO with the same precision as the original cataract procedure. The advances outlined here mark key steps toward a future in which PCO management is not only more effective and efficient but also customized to each patient’s needs, ultimately preserving vision and quality of life.
